# Factors Affecting the Decision Regarding COVID-19 Vaccination in the Saudi Public in the Central Region

**DOI:** 10.7759/cureus.28874

**Published:** 2022-09-07

**Authors:** Adel F Almutairi, Yousef M Alessa, Shoug Alhizam, Hana Aljabri, Alanood Algharibi, Suraia Enizi, Ala'a BaniMustafa

**Affiliations:** 1 Research Performance and Outcome Department, King Abdullah International Medical Research Center, King Saud bin Abdulaziz University for Health Sciences, Ministry of National Guard-Health Affairs, Riyadh, SAU

**Keywords:** myths, vaccine acceptance, hesitancy, vaccination, covid-19

## Abstract

Introduction: In response to the disease, multiple companies created coronavirus disease 2019 (COVID-19) vaccinations. These vaccines were developed utilizing a variety of technologies and at an unprecedented rate, leading many people to question their efficacy and safety, as well as what they thought about how well the vaccination may protect them. As a result, the goal of this study was to evaluate the factors and motivators that may affect the Saudi Arabian population's decision to get COVID-19 vaccination.

Methods: A sample of Saudi citizens from the Central Region completed an electronic questionnaire. This questionnaire assessed a variety of factors, including why people choose to get or not have the COVID-19 vaccination.

Results: In total, 526 Saudis responded to the survey, with the average age being 35±11 years. Of the participants, 408 (77.6%) had received COVID-19 vaccination (one or two doses), and 118 (22.4%) had not been vaccinated. Females (n=233, 73%, P=0.002) and the group less than 35 years (n=223, 54.7%, P=0.017) were more likely than the males to get vaccinated. Married (n=256, 80.5%) and employed (n=261, 81.1%) participants had higher vaccination rates than unmarried and unemployed. Major reasons for not being vaccinated were a lack of knowledge about the adverse effects (n=74, 62.7%), concerns about possible side effects (n=70, 59.3%), and a lack of faith in the vaccination (n=45, 38.1%). Receiving flu vaccination was significantly associated with being vaccinated against COVID-19 (P=0.020).

Conclusion: Lack of knowledge about the vaccine's side effects and uncertainty were the major deterrents to vaccination, whereas faith in the Ministry of Health's instructions was the key motivator.

## Introduction

Within a month of its onset, the latest coronavirus disseminating a global challenge, as there is no specific antiviral treatment for coronavirus disease 2019 (COVID-19). In the absence of a vaccine, countries contained the spread of COVID-19 through quarantine and lockdown, social distancing measures, community-wide use of facemasks at all times, and travel bans. These measures caused significant impairment to people's physical and psychosocial well-being, as well as a massive reduction in the global economy [1,2]. Vaccination was important to reduce the spread of the infection. Several global studies reported the importance of vaccination in preventing many different diseases, and the production of a vaccine against COVID-19 accelerated at an exponential rate to minimize and control the pandemic [3,4].

In an effort to combat the prevalence of COVID-19, many governments granted an Emergency Use Authorization (EUA) to FDAs that allowed using COVID-19 vaccines prior to being approved [5,6]. In addition, the World Health Organization (WHO) provided an Emergency Use Listing (EUL) of additional vaccines after the declaration of the public health emergency. Because of the EUA and EUL, multiple COVID-19 vaccines have become authorized and recommended for distribution and utilization [7]. The COVID-19 vaccines include the Pfizer-BioNTech, Moderna, Johnson & Johnson’s Janssen, Oxford-AstraZeneca, Sinovac, Sinopharm, and Serum Institute of India Pty Ltd [8,9]. These vaccines were developed in several countries, each with a different efficacy rate. Preliminary studies indicated that the efficacy rate of the messenger ribonucleic acid (mRNA) vaccine technology, produced by Pfizer and Moderna, is more than 90%, whereas Oxford-AstraZeneca and Johnson & Johnson’s Janssen have efficacy rates of 76% and 72%, respectively [10].

Vaccine hesitancy is a serious public health concern, and the vaccination uptake rates in Middle Eastern countries vary greatly, depending on region and time of the year [1,11]. Three major causes are ascribed to vaccine hesitancy: (i) individuals' lack of confidence in and fear of vaccines, especially if they believe that the vaccines pose a risk of infection; (ii) individuals not experiencing the need for a vaccine or do not trust the vaccine source; and (iii) individuals or communities have difficulty receiving the vaccine [12]. Several studies identified a number of factors that influence the acceptance of a new vaccine. These factors are related to individuals’ beliefs about the vaccine's protection and effectiveness, negative health effects, myths regarding the need for vaccination, a lack of interest in the health system, and a lack of community awareness about vaccine-preventable diseases [13].

In Saudi Arabia, the vaccine against COVID-19 was available soon after it was released. The Saudi Ministry of Health accredited only two COVID-19 vaccines, namely Pfizer-BioNTech and AstraZeneca [14]. The recommended vaccine dose, for both vaccines, is two doses. The Ministry of Health conducted large media campaigns to increase awareness of the importance of the vaccine in order to safeguard the Saudi population and help people get back to their normal life. As of August 31, 2021, 62% of the population received at least one dose, and 41% of the population is fully vaccinated [15]. Yet, COVID-19 vaccine acceptance and hesitancy are a concern for many people, and this was the main driver to conduct the current study, which aimed to investigate the factors and motivators that would affect the decision of the Saudi Arabian population regarding vaccination. 

## Materials and methods

Population and sampling

This cross-sectional study was conducted on a sample of the general public in the Central Region of Saudi Arabia. Adult Saudi nationals (18 years or older) were eligible for participation. Using a snowball technique, the survey link and initiation were disseminated to the public electronically, and those who received it were asked to share it with their network of contacts. In Saudi Arabia, social media is a key medium for communication and discussion, and it is regarded as a potent instrument for effective recruitment, as the invitation to participate may reach a large number of individuals in a short period. The letter of invitation, describing the objectives and potential outcomes, and the participants can choose to refuse or participate through completing the form.

As the primary objective of the study was to identify the factors influencing the public’s decision to be vaccinated, we assumed the prevalence of vaccine hesitancy as 0.5 (as no information is available on proportion), the expected sample size was 385. This estimation had a 95% confidence level (z=1.96), and a margin of error (precision) of 0.05. in order to account for the possibility of a low response rate, we oversampled by 40% of the expected sample size resulting in a final sample size of 532.

Data collection

The data was collected using a self-administered questionnaire measuring several variables, such as the reasons that supported the decision to be vaccinated or not be vaccinated. The questionnaire was comprised of three sections. The first section covered the socio-demographic characteristics, including age, gender, marital status, work status, area of residence, and education level. The second section, which contained 15 items, focused on the factors that influenced a positive decision to be vaccinated and the third section was about myths and concerns about the COVID-19 vaccine. These items were developed, following an extensive literature review [2,16,17], and consultation with a number of public health experts. The responses were yes and no options. The questionnaire was translated to Arabic by the research team and then back-translated to English to ensure accuracy.

Data management and analysis plan

The data were analyzed using the Statistical Package for Social Sciences (SPSS) version 27 (IBM Corp., Armonk, NY, USA). Descriptive statistics such as the mean score and standard deviation, as well as frequency and percentage were used to present the data. All the items were tabulated and compared between the groups in terms of the demographic variables, for example age, gender, working specialty, and marital status using the Chi-square test. Significance was considered at a p-value <0.05.

Ethical considerations

The Institutional Review Board of the Ministry of National Guard-Health Affairs provided ethical clearance and approval for this study (RC16/09). The questionnaire had no self-identifiers, and participation was optional and anonymous.

## Results

Participant demographic information

The sample size was realized as 526 participants. The majority were female (n=319, 60.6%) in different age groups, and the mean age was 35±11 years. More than half of the sample (n=322, 61.2%) were employed, of which 162 (30.8%) were working in healthcare. The majority (63.5%, n=334) had an undergraduate degree or school level education, with 19.8% having a postgraduate degree. The majority (82.3%, n=433) were non-smokers, and 85 (16.2%) reported having a chronic disease. A high proportion (77.6%, n=408) received one or two doses of a COVID-19 vaccine and 121 (23%) were previously infected. Of the sample, 421 (80%) said they knew someone who had the COVID-19 infection who was a family or close friend. Table 1 presents more details on the socio-demographic profile of the study subjects.

**Table 1:** Participants' characteristics

Sample Size	N = 526
Gender	
Male	207 (39.4%)
Female	319 (60.6%)
Age	
≤ 35	302 (57.4%)
˃ 35	224 (42.6%)
Mean SD	35±11
Educational Level	
School	88 (16.7)
Undergraduate	334 (63.5)
Postgraduate	104 (19.8)
Marital Status	
Married	318 (60.5%)
Unmarried	208 (39.5%)
Work Status	
Employed	322 (61.2%)
Unemployed	204 (38.8%)
Healthcare Worker	
Yes	162 (30.8%)
No	364 (69.2%)
BMI	
Normal	205 (39.2%)
Over weight	183 (35%)
Obesity	135 (25.8%)
Chronic Diseases	
Yes	85 (16.2%)
No	441 (83.8%)
Smoker	
Yes	93 (17.7%)
No	433 (82.3%)
Previously Infected with COVID-19	
Yes	121 (23%)
No	405 (77%)
Infected with COVID-19 (Relative or close friend)	
Yes	421 (80%)
No	105 (20%)
Flu Vaccine	
Yes	122 (23.2%)
No	404 (76.8%)
COVID-19 Vaccine	
Yes	408 (77.6%)
No	118 (22.4%)
Type of the Vaccine Pfizer	299 (73.3%)
AstraZeneca	104 (25.5%)
Sinopharm	5 (1.2%)

Myths and concerns about COVID-19 vaccination

All the statements used were negative beliefs about the vaccine. The results were divided into a non-vaccinated group (n=118, 22.4%) and a vaccinated group (n=408, 77.6%). For the non-vaccinated group, 49 (41.5%) reported not believing that the vaccine is safe, 34 (28.8%) thought the virus was developed to make money, a small proportion (15, 12.7%) thought that the vaccine will alter their genetic background, and 17 (14.4%) believed that the vaccination aimed to introduce nano-chips in their body. The majority of the vaccine group disagreed with all the statements related to myths regarding the COVID-19 vaccine; 385 (94.4%) disagreed that the vaccine will introduce nano-chips in their body and 378 (92.6%) disagreed that the vaccine will infect them with COVID-19. However, 86 (21.1%) agreed that the virus was developed for financial gain. Table 2 illustrates the myth and concerns that might influence the decision to be vaccinated.

**Table 2:** Myths and concerns about the COVID-19 vaccine

Myths and Concerns	Group 1 (Not vaccinated) N=118		Group 2 (Vaccinated) N=408	
	Agreed	Disagreed	Agreed	Disagreed
I think that covid19 is a hoax	22(18.6%)	96(81.4%)	46(11.3%)	362(88.7%)
I think covid19 vaccine will alter my DNA (genetic background)	15(12.7%)	103(87.3%)	41(10%)	367(90%)
I believe that Covid‐19 vaccine can affect women fertility	23(19.5%)	95(80.5%)	33(8.1%)	375(91.9%)
The virus’s spread is part of a global plan to reduce the world’s population	27(22.9%)	91(77.1%)	59(14.5%)	349(85.5%)
The virus was developed and spread around the world by certain people for money making	34(28.8%)	84(71.2%)	86(21.1%)	322(78.9%)
covid19 vaccine will implement nano-chips in my body	17(14.4%)	101(85.6%)	23(5.6%)	385(94.4%)
I don’t believe the vaccine is safe	49(41.5%)	69(58.5%)	57(14%)	351(86%)
I think the vaccine shot will make me infected with covid19	30(25.4%)	88(74.6%)	30(7.4%)	378(92.6%)

Reasons for and against the vaccine

Lack of knowledge about the adverse effects (62.7%, n=74), worry about possible side effects (59.3%, n=70), and lack of faith in the vaccination (38.1%, n=45) were the main three reasons given for not getting the vaccine. In the vaccinated group, the major reason for being vaccinated was related to the fear of infecting their family members (n=345, 84.6%), followed by the Ministry of Health recommendations (n=325, 79.7%), travel requirements (n=245, 60%), fear of being infected with COVID‐19 (n=236, 57.8%), as well as family/friends recommendations (n=203, 49.8%). The lowest scoring statements were related to feeling embarrassed if they were not vaccinated (n=33, 8.1%), and job requirements (n=131, 32.1%). Table 3 presents more details regarding the reasons for taking the vaccine.

**Table 3:** Reasons that influenced participants to take the vaccine

Reason for taking the vaccine N=408	Yes	No
Recommendations of the Physician	150 (36.8%)	258 (63.2%)
Recommendations of the Family/Friends	203 (49.8%)	258 (63.2%)
Recommendations of the Ministry of Health	325 (79.7%)	83 (20.3%)
Job required	131 (32.1%)	277 (67.9%)
Hajj/Omra	142 (34.8%)	266 (65.2%)
Travel requirements	245 (60%)	163 (40%)
Fear of being infected with COVID‐19	236 (57.8%)	172 (42.2%)
Fear of infecting my family, especially my parents	345 (84.6%)	63 (15.4%)
Availability of free vaccines	317 (77.7)	91 (22.3%)
Feeling embarrassed if I didn’t take the vaccine	33 (8.1%)	375 (91.9%)
Reason for not taking the vaccine N=118	Yes	No
Lack of information about the vaccine	33 (8.1%)	375 (91.9%)
Lack of information about the vaccine side effects	74 (62.7%)	44 (73.3%)
Don’t trust the vaccine	45 (38.1%)	45 (38.1%)
Worried about the potential side effects	70 (59.3%)	48 (40.7%)
Fear of injections	19 (16.1%)	99 (83.9%)
Avoid the medical products	37 (31.4%)	81 (68.6%)
Lack of time	22 (18.6%)	96 (81.4%)

Factors associated with COVID-19 vs. flu vaccination

In terms of the factors associated with receiving a COVID-19 or flu vaccine, the study findings showed that the males were more likely than the females to receive the vaccine (84.5%, n=175), with a significant gender difference (P=0.002), other factors included age over 35 years old (82.6%, n=185, P=0.017), being married (80.5%, n=256, P=0.046), employed (81.1%, n=261, P=0.016), smoker (87.1%, n=81, P=0.015), and never infected with COVID-19 (82.5%, n=334, P<0.001). The group with a postgraduate degree had the highest proportion of being vaccinated (82.7%, n=86, P=0.012), of which 32.7% (n=34, P=0.012) also received a flu vaccine, 91 (28.3%, P<0.001) were employed and 62 (38.3%, P<0.001) were a healthcare worker. Being vaccinated with a flu vaccine was significantly associated with vaccination with COVID-19 (P=0.020) (Table 4).

**Table 4:** Relation between participants' characteristics and receipt of COVID-19 or flu vaccine

Demographic	FLU Vaccine								COVID19 Vaccine	
	Yes				no				Yes	No
Gender:										
Male	55(26.6%)				152(73.4)				175(84.5%)	32(15.5%)
Female	67(21%)				252(79%)				233(73%)	86(27%)
	χ2= 2.184, P= .139								χ2= 9.542 , P= 0.002	
Age:										
≤35 ≥35	77 (25.5%)			225 (74.5%)					223 (73.8%)	79 (26.2%)
	45 (20.1%)			179 (79.9%)					185 (82.6%)	39 (17.4%)
	χ2= 2.111 , P= 0.146								χ2= 5.656 , P= 0.017	
Education:										
School	13 (14.8%)			75 (85.2%)					58 (65.9%)	30 (34.1%)
Undergraduate	75 (22.5%)			259 (77.5%)					264 (79%)	70 (21%)
Postgraduate	34 (32.7%)			70 (67.3%)					86 (82.7%)	18 (17.3%)
	χ2= 8.873 , P= 0.012								χ2= 8.861 , P= 0.012	
Marital:										
Married	76 (23.9%)		242 (76.1%)						256 (80.5%)	62 (19.5%)
Unmarried	46 (22.1%)		162 (77.9%)						152 (73.1%)	56 (26.9%)
	χ2= 0.225 , P= 0.636								χ2= 3.985 , P= 0.046	
Occupation Status”										
Unemployed	31 (15.2%)					173 (84.8%)			147 (72.1%)	57 (27.9%)
Employed	91 (28.3%)					231 (71.7%)			261 (81.1%)	61 (18.9%)
	χ2= 11.966 , P= <0.001								χ2= 5.809 , P= 0.016	
Healthcare worker:										
Yes	62 (38.3%)					100 (61.7%)			133 (82.1%)	29 (17.9%)
No	60 (16.5%)					304 (83.5%)			275 (75.5%)	89 (24.5%)
	χ2= 29.874 , P= <0.001								χ2= 2.763 , P= 0.096	
BMI Classification										
Normal	43 (21%)							162 (79%)	157 (76.6%)	48 (23.4%)
Overweight	45 (24.6%)							138 (75.4%)	142 (77.6%)	41 (22.4%)
Obesity	34 (25.2%)							101 (74.8%)	107 (79.3%)	28 (20.7%)
	χ2= 1.058 , P= 0.589								χ2= 0.335 , P= 0.846	
Chronic Diseases										
Yes	21 (24.7%)				64 (75.3%)				64 (75.3%)	21 (24.7%)
No	101 (22.9%)				340 (77.1%)				334 (78%)	97 (22%)
	χ2= 0.130 , P= 0.718								χ2= 0.301 , P= 0.583	
Smoker										
Yes	22 (23.7&)	71 (76.3%)							81 (87.1%)	12 (12.9%)
No	100 (23.1%)	333 (76.9%)							327 (75.5%)	106 (24.5%)
	χ2= 0.014 , P= 0.907								χ2= 5.897 , P= 0.015	
Previous infection with COVID19										
Yes	23 (19%)	98 (81%)							74 (61.2%)	47 (38.8%)
No	99 (24.4%)	306 (75.6%)							334 (82.5%)	71 (17.5%)
	χ2= 1.546 , P= 0.214								χ2= 24.319 , P= <0.001	
family or friends infected with COVID19										
Yes	104 (24.7%)						317 (75.3%)		331 (78.6%)	90 (21.4%)
No	18 (17.1%)						87 (82.9%)		77 (73.3%)	28 (26.7%)
	χ2= 2.696 , P= 0.101								χ2= 1.351 , P= 0.245	
Take flu vaccine										
Yes									104 (85.2%)	18 (14.8%)
No									304 (75.2%)	100 (24.8%)
									χ2= 5.383 , P= 0.020	

## Discussion

Fear of vaccines is an obstacle in the global attempt to control the current pandemic, which has negatively affected individuals’ health and the economy. Understanding the factors that prevent or encourage people to be vaccinated, is critical for planning and accelerating the vaccination process. This study identified several factors associated with the decision regarding vaccination, including that males were more likely than females to receive the COVID-19 vaccination. This finding is consistent with the studies from Egypt and Portugal, which illustrated that females had lower compliance with vaccination [2,18].

The current study indicated that 38% of the non-vaccinated group did not trust the vaccines and 41.5% did not believe that the vaccine is safe, despite the fact that the majority disagreed with the statement of lacking information about the vaccine (91.9%) and its side effects (73.3%). An explanation for this inconsistency is that individuals believe they have sufficient understanding of the new vaccinations, which is compounded by media misinformation. Many studies also indicated that anti-vaccination messages posted by very active vaccine-hesitant groups [19] might influence people negatively to decline the vaccine and increase their hesitancy, as well as the widespread misinformation during the pandemic [19]. As a result, to persuade populations to adopt vaccination, solid proof of vaccine safety and efficacy, supported by clinical trial findings, is required.

The current study identified several factors associated with the willingness to be vaccinated, including gender, age, and educational level. This result is consistent with a study about vaccine acceptance, in which older people and males were more compliant with vaccination [20]. Vaccination rates were higher in the group with a higher educational level, this might be explained by their lower proclivity to believe in conspiracies [20]. The married group was more likely to take the COVID-19 vaccine, possibly due to their fear of infecting their beloved family members. This point was supported by our study which indicated that 81.7% who received the vaccine were due to their fear of infecting their families. The employed group was also more likely to be vaccinated, a mandatory job requirement in the Kingdom of Saudi Arabia. Being a smoker was associated with higher rate of being vaccinated, possibly due to the proportion of male smokers, which is higher than female smokers in Saudi Arabia [21].

Participants who reported that they were previously infected with COVID-19 had a higher rate of vaccination which might be explained by their level of knowledge regarding the seriousness of the disease itself, as well as their experience with the disease. Our findings also revealed that participants who received the flu vaccine were more likely to receive the COVID-19 vaccine. This finding could be explained by the fact that individuals who are more protective against the general flu are also more protective against other types of respiratory disease; however, no study was found to support this theory. A study conducted in Saudi Arabia with healthcare workers [22] indicated that 76.76% of the group who accepted the COVID-19 vaccination had previously received the seasonal influenza vaccine, which might explain the association between the two vaccines.

Our study also assessed the factors that could positively influence a person's decision to get the flu vaccine, with the goal of determining the intensity of the impact and to compare the impact of same factors regarding the COVID-19 vaccine. We found that the education level and employment status influenced the decision to have the flu and the COVID-19 vaccine. These two variables may be the main influencing factors affecting vaccination against respiratory diseases. The comparison in our study is weak due to the difference in the severity of the consequences if the individual was not vaccinated. We believe that an additional study is required to establish the strength of these factors and their influence on public vaccination acceptability, which might aid decision-makers in determining which group should be given priority.

This study has a number of limitations. The study sample is limited to the perspectives of the people who are living in the Central Region of Saudi Arabia, which may affect the generalizability elsewhere. In addition, the survey was limited to individuals who are able to use social media and access the link to the electronic questionnaire. The sample size was relatively small and this might be due to the online sampling within a short time, as well as the frequent exposure of individuals to a great number of studies during the pandemic. This could have affected their interest to participate in a new study. Additional studies are recommended, with qualitative methods, to explore the reasons and motives of people to be vaccinated, and provide a deep understanding of their concerns.

## Conclusions

The study findings are important as they highlight the major reasons for not being vaccinated against COVID-19, as well as the motivators for vaccination in Saudi Arabia's central region. The main reason for not being vaccinated was lack of information about the side effects of the vaccine and uncertainty, and the major positive reason for vaccination was the trust in the Ministry of Health recommendations. However, the study also revealed a lack of trust about the vaccine's efficacy and safety. The factors that were significantly linked with a higher rate of COVID-19 vaccination were being male, 35 years of age or older, having a postgraduate degree, being employed and prior COVID-19 infection. Factors that influence the decision to have the flu vaccine do not necessarily affect the decision to have the COVID-19 vaccine, however, the educational level and employment status are factors related to a positive decision for both vaccines. It is important to design effective strategies to promote the COVID-19 vaccine uptake in females, young individuals, and less educated or unemployed individuals. 
